# Combination therapy in hypertension: An update

**DOI:** 10.1186/1758-5996-2-44

**Published:** 2010-06-24

**Authors:** Sanjay Kalra, Bharti Kalra, Navneet Agrawal

**Affiliations:** 1 Dept of Endocrinology, Bharti Hospital, Karnal, India; 2 Dept of Gynaecology, Bharti Hospital, Karnal, India; 3 Dept of Medicine, GR Medical College, Gwalior, India

## Abstract

Meticulous control of blood pressure is required in patients with hypertension to produce the maximum reduction in clinical cardiovascular end points, especially in patients with comorbidities like diabetes mellitus where more aggressive blood pressure lowering might be beneficial. Recent clinical trials suggest that the approach of using monotherapy for the control of hypertension is not likely to be successful in most patients. Combination therapy may be theoretically favored by the fact that multiple factors contribute to hypertension, and achieving control of blood pressure with single agent acting through one particular mechanism may not be possible. Regimens can either be fixed dose combinations or drugs added sequentially one after other. Combining the drugs makes them available in a convenient dosing format, lower the dose of individual component, thus, reducing the side effects and improving compliance. Classes of antihypertensive agents which have been commonly used are angiotensin receptor blockers, thiazide diuretics, beta and alpha blockers, calcium antagonists and angiotensin-converting enzyme inhibitors. Thiazide diuretics and calcium channel blockers are effective, as well as combinations that include renin-angiotensin-aldosterone system blockers, in reducing BP. The majority of currently available fixed-dose combinations are diuretic-based. Combinations may be individualized according to the presence of comorbidities like diabetes mellitus, chronic renal failure, heart failure, thyroid disorders and for special population groups like elderly and pregnant females.

## Review

Achieving recommended goal of blood pressure (BP) {< 140/90 mmHg in all hypertensives, < 130/80 mm Hg in hypertensives with diabetes mellitus (DM) [1]} is difficult in majority of patients with hypertension [2]. Various studies have shown that tight control of BP is required to produce the maximum reduction in clinical cardiovascular end points [3,4]. The Framingham Heart Study[5] indicated that a 2-mm Hg reduction in average diastolic blood pressure (DBP) could result in a 14% decrease in the risk of stroke and transient ischemic attacks and a 6% reduction in the risk of coronary artery disease. A meta-analysis of 9 major prospective observational studies also showed that prolonged reduction in DBP of 5, 7.5, and 10 mm Hg were associated with 34%, 46%, and 56% fewer strokes and 21%, 29%, and 37% lower incidences of coronary heart disease respectively [6]. These data suggest that more aggressive BP lowering might be beneficial. Though single drug treatment may be effective in some, more than 50% will require more than one drug for appropriate control of their BP.

The Seventh Report of the Joint National Committee on Prevention, Detection, Evaluation, and Treatment of High Blood Pressure (JNC 7) and European Society of Hypertension (ESH) guidelines recommend that therapy with more than one antihypertensive agent be considered in patients with systolic blood pressure (SBP) more than 20 mm Hg or DBP more than 10 mm Hg above goal and among patients at high cardiovascular risk, as determined by elevated BP level and the presence of other risk factors [7,8]. The approach of combination therapy may be theoretically favored by the fact that multiple factors contribute to the hypertension and achieving control of BP with single agent that acts through one particular mechanism may be unrealistic. Combining the second agent may lead to better control, acting by complimentary mechanism.

This review focuses the need and basis of combination therapy, different classes of combination agents available at present, rationale for their combination, comparisons of these combinations and their effect on the outcome.

### Basis of combination therapy

National Harris interactive survey for hypertension, in the United States revealed that out of 90% patients taking medication only 50% to 60% were involved in some form of lifestyle change to control BP [9]. Thus majority of patients with hypertension rely on medication for the control of their BP. More recent clinical trials suggest that the approach of using monotherapy for the control of hypertension is not likely to be successful in most patients and specially in those with some comorbidities (eg. DM, heart failure). The achievement of BP goal typically require 2 or more medications in various settings [10-14]. For instance, [15] in a factorial study with 1461 patients randomized to 16 treatment groups, taking telmisartan 0, 20, 40, 80 mg and amlodipine 0, 2.5, 5, 10 mg for 8 weeks, greater BP reductions were observed with combination therapy than with respective monotherapies. Highest dose combination (telmisartan 80 mg plus amlodipine 10 mg) had the greatest least square mean systolic/diastolic BP reductions (26.4/20.1 mm Hg; P < 0.05 compared with both monotherapies) with over 90% BP response rates. Peripheral edema was most common in the amlodipine 10-mg group (17.8%) but the rate had notably lowered when amlodipine was used in combination with telmisartan. Similar results were observed with other trial of olmesartan medoxomil/amlodipine combination therapy vs. respective monotherapies where more effective BP reduction and BP goals (44.5-54% vs 28.5-30%) were achieved with combination therapy than with either of monotherapies. Over 70% of patients on combination therapy achieved BP goals [16].

Another double blind, parallel group randomized study for 12 weeks comparing the combination therapy of felodipine and metoprolol (5/50mg) with either monotherapy exhibited significantly greater antihypertensive response (98%) with combination compared to monotherapy (felodipine- 79% and metoprolol- 82%). A significant greater reduction in mean systolic/diastolic BP (28/18 mmHg) with combination therapy was evinced compared to either felodipine (18/12 mm hg) or metoprolol (19/12 mm hg) [17].

The long-term (I year) efficacy and safety between lisinopril and trichlormethazide combination therapy and lisinopril monotherapy was investigated in a multi-centre open label trial on 466 patients. It showed effective BP reduction to < 150/90 mmHg in both the groups throughout the study period. Additionally the combination of trichlormethazide reversed the increase in serum potassium observed in the monotherapy group [18].

The results of these studies emphasize that multiple-drug therapy was both safe and effective compared to monotherapy and will be required in most patients to attain BP goals.

Many panels including Hypertension in African Americans Working Group (HAAWG), JNC 7, The Task Force for the Management of arterial Hypertension of the ESH and of the European Society of Cardiology have strongly supported that treatment initiation with 2 or if needed 3 drugs is justified in many cases of hypertension management [7-14,19]. There are other various advantages with combination therapy. Combining the drugs makes them available in a convenient dosing format, lowers the dose and can be given in once daily schedule thus improving compliance. There is an additive or synergistic antihypertensive effect at lower doses of individual components and at the same time the drugs in combination counteract the side effects of each other. This helps more patients to achieve normal BP and even can be effective in hard-to-treat populations. Early normalization of BP may greatly motivate the patients to adhere to lifelong treatment.

### Available options in combination therapy

Multi-drug therapy regimens can either be fixed dose combinations (FDCs) or drugs added sequentially one after other. However, choice of combination antihypertensive therapy will depend upon the tolerability and a convenience of dosing/titrating drug regimen. FDCs can enhance adherence to medication regimens compared with treatment given as 2 separate agents. Also they facilitate more prompt reduction in BP. The use of antihypertensive combinations started in the 1960s with hydrochlorothiazide (HCTZ) combined with triamterene, a potassium-sparing diuretic, and has been added with newer and different combinations in due course of time [20].

Available trials have studied different classes of drugs in combination for treatment of hypertension, taking advantage of their complimentary action. Angiotensin receptor blockers (ARBs), thiazide diuretics, alpha and beta blockers, calcium antagonists(CCBs) and angiotensin-converting enzyme inhibitors (ACEIs) have been the commonly used classes of antihypertensive agents. Thiazide diuretics and CCBs are effective, as well as combinations that include rennin-angiotensin-aldosterone system (RAAS) blockers, in reducing BP. Several combinations of an ACEI or ARB with a diuretic or an ACEI with a CCB are available. The majority of currently available FDCs are diuretic-based. Though diuretics have an unparalleled track record of safety and efficacy, recent data documenting low-grade carcinogenicity must be evaluated further [21].

Some of the commonly available combinations are listed in Table 1

**Table 1:** Pharmacological rationale of combination therapy

Combinations	Mechanisms	
**ARB-Diuretic**	ARBs cause the antagonism of angiotensin II at the vascular and myocardial level by direct AT-1 receptor blockade	Thiazide diuretic blocks sodium chloride reabsorption at the distal convoluted tubule

**β-Adrenoceptor Antagonist-Diuretic**	The β-adrenoceptor blocker inhibits activation by direct suppression of renin release, inhibit β-adrenergic sympathetic stimulation decreasing myocardial contractility and heart rate	Diuretics as above

**ACEI-Diuretic**	ACEI cause the removal of the angiotensin II effect (vasoconstriction, stimulation of aldosterone secretion) and enhancement of kinin-mediated vasodilation	Diuretics as above

**ACEI-CCB**	ACEI as above	The calcium antagonists de-crease vascular resistance by vascular smooth muscle relaxation

**ARB-CCB**	ARBs as above	CCBs as Above

**ACE-ARB Inhibitors**	ACEI as above	ARBs as above

**Centrally Acting Agents-Diuretic**	Clonidine acts by decreasing sympathetic outflow by stimulating pre synaptic α_2_-adrenoceptors in the vasomotor centre of the CNS.	Diuretics as above

Angiotensin Converting Enzyme (ACE) inhibitors, Angiotensin II type 1 Receptor Blockers (ARBs), Calcium Channel Antagonist (CCB)

Some of the combinations which have been studied in detail are discussed below. Refer Table 2 for commonly available FDCs.

**Table 2:** Fixed- dose combinations with examples

Combinations	Fixed dose combinations examples
**ARB-Diuretic**	Irbesartan/HCTZ
	Losartan/HCTZ
	Telmisartan/HCTZ
	Valsartan/HCTZ

**β-Adrenoceptor Antagonist-Diuretic**	Atenolol/chlortalidone
	Metoprolol/HCTZ
	Propranolol/HCTZ

**ACEI- Diuretic**	Captopril/HCTZ
	Enalapril/HCTZ
	Lisinopril/HCTZ
	Moexipril/HCTZ

**ACEI- CCB**	Benazepril/Amlodipine
	Trandolapril/Verapamil

**ARB-CCB**	Amlodipine/Olmesartan medoxomil
	Amlodipine/Valsartan
	Amlodopine/Telmisartan

**ARB-ACEIs**	Telmisartan/Ramipril

**Centrally Acting Agents-Diuretic**	Clonidine/Chlortalidone

Angiotensin Converting Enzyme (ACE) inhibitors, Angiotensin II type 1 Receptor Blockers (ARBs), Calcium Channel Antagonist (CCB), hydrochlorothiazide (HCTZ), renin- angiotensin-aldosterone system (RAAS)

### β Blockers with diuretics

β-blockers and diuretics have been used for the treatment of hypertension for more than three decades. Although β-blockers did have a beneficial effect on the BP, β-blocker therapy failed to favourably affect the cardiovascular events and mortality either alone or in combination with a diuretic [22]. Warmack reviewed and evaluated 5 placebo- controlled studies, 10 active-controlled studies and 11 meta-analyses for assessing the effects of β-blockers on cardiovascular and cerebrovascular outcomes in the treatment of hypertension. Most of the studies included atenolol and the combination drug often used was thiazide diuretic. β-blockers showed increased risk for stroke, cardiovascular events and mortality in majority of the studies as compared to other anti-hypertensives. Only 2 comparison studies of β-blockers evidenced significant cardiovascular benefit [23].

Earlier it was a widely held belief that beta-blockers should be prescribed for management of hypertension in patients with higher heart rates, an established risk factor for cardiovascular events. But recent Anglo-Scandinavian Cardiac Outcomes Trial-Blood Pressure Lowering Arm (ASCOT-BPLA) trial concluded that, in similar hypertensive populations without previous or current coronary artery disease, higher baseline heart rate is not an indication for preferential use of β-blocker-based therapy over amlodipine based therapy. ASCOT BP-lowering arm showed outcome inferiority of therapy initiated with atenolol versus that initiated with CCB, amlodipine (including mortality disadvantage) [24]. Also the risk of sudden cardiac death was found to be higher in elderly patients receiving either b-blockers as monotherapy, or in combination with a thiazide diuretic, than in patients receiving another form of therapy (CCBs, , or potassium-sparing diuretics) [25].

So based on above evidence beta-blockers alone or in combination should now have more restricted place in cardiovascular therapy and can be possibly indicated in hypertensives with anxiety and fast heart rate.

### ACEIs/ARBs with Diuretics

RAAS inhibitor and a diuretic combination will offset the diuretic-induced increase in plasma renin activity. The salt loss will add to the antihypertensive effect of RAAS blocker. Besides, an ARB will also attenuate the metabolic effects of thiazide diuretics like hypokalemia and hyperglycemia. Several studies have demonstrated the antihypertensive effectiveness of this combination in low doses, showing substantially greater reductions in BP and higher response rates than either of the treatments alone [26,27].

The Action in Diabetes and Vascular Disease: Preterax and Diamicron MR Controlled Evaluation (ADVANCE), trial compared the effects of BP lowering with a perindopril/indapamide combination or placebo, in high-risk type 2 diabetic subjects. The risk of combined primary outcome, a major macrovascular or microvascular event was reduced by 9% (p = 0.041) with a 14% (p = 0.025) reduction in all-cause mortality and an 18% (p = 0.027) reduction in cardiovascular mortality. The study extrapolated saving one death over 5 years for every 79 patients with this ACEI/Diuretic combination [28]. Similarly clinical studies have shown combination of the ARB, irbesartan with HCTZ to be safe and effective in patients with moderate to severe hypertension, irrespective of baseline BP level, age, obesity, race, diabetic status, and the metabolic syndrome and has a significantly greater dose-dependent BP lowering effect than either agent alone [29,30]. One study suggested that although a goal BP of < 140/90 mm Hg can be reached in the majority of patients with SBP < 160 mm Hg with irbesartan monotherapy, most patients with moderate to severe (stage 27 or grade 2 or 3) hypertension (baseline SBP ≥ 160 mm Hg) require combination therapy for BP goal to be reached [31,32].

A trial with patients having uncontrolled BP despite antihypertensive agents including an ARB (candesartan 8 mg/day or valsartan 80 mg/day) randomly assigned to combination therapy with telmisartan 40 mg/day and HCTZ 12.5 mg/day (T + H, n = 32) or to no change in their current drug regimen (n = 32). Both office and home BP was significantly reduced in T+H arm in 12 weeks. Also early morning BP was decreased inferring the long duration activity of combination [33]. ONEAST study also showed significant BP reduction in telmisartan+amlodipine group than amlodipine group alone (decrease in BP: -9.9+/-11.4 vs. -3.7+/-8.9 mm Hg, P < 0.02; normalization rate: 67.6 vs. 30.3%, P < 0.01). Thus it seems that the combination of a RAAS blocker and a low-dose thiazide is useful if treatment with a CCB cannot control BP in patients with hypertension [34]. The results of these studies confirm that diuretic/ACEI or diuretic/ARB combinations reduce BP further than monotherapies in hypertensive diabetic subjects with an acceptable safety profile.

### RAAS Blocker with CCB

RAAS blocker buffer CCB-induced activation of the sympathetic nervous system and the RAAS. Also the negative sodium balance caused by CCBs adds to the antihypertensive effect of RAAS blocker. Dose-dependent CCB induced peripheral edema may be minimized in the presence of an RAAS blocker [35].

### ACEIs with CCB

In patients with both diabetes and hypertension, ACEIs provide clinical benefits that appear to be independent of BP reduction [36]. In the Fosinopril vs Amlodipine Cardiovascular Events Trial (FACET) [37] in patients with hypertension and diabetes those receiving fosinopril were approximately 50% less likely to experience a major cardiovascular event than those receiving amlodipine when followed for up to 3.5 years. The number of observed vascular events was even lower in those who received combination. Similarly in Effects of Antihypertensive Agents on Cardiovascular Events in Patients With Coronary Disease and Normal Blood Pressure (CAMELOT) [38] 2 years of treatment with amlodipine significantly reduced the incidence of CV adverse events. The ANDI study demonstrated that in hypertensive patients with diabetes whose BP was not controlled with 20 mg quinapril alone, initiation of combination therapy by adding 5 mg amlodipine besylate to quinapril 20 mg was more effective in reducing BP than increasing the dose of quinapril to 40 mg [39].

### Combination ARB with CCB

The rationale for combination therapy with agents that block the RAAS and a CCB or diuretic is well founded [40]. However, the use of ARBs and CCBs has independent benefits beyond BP lowering, on morbidity and mortality in patients with hypertension and comorbid conditions. In the Losartan Intervention for Endpoint Reduction in Hypertension (LIFE) study, a losartan-based (ARB) regimen significantly reduced the relative risk of cardiovascular-related morbidity and death in hypertensive patients with left ventricular hypertrophy by 13% (*P *= .02). The reduction came, however, mostly as a result of a 25% reduction in the relative risk of stroke (*P *= .001), compared with atenolol-based therapy, yet the between-group difference in SBP was only 1 mm Hg [41]. Moreover telmisartan has a different pharmacokinetic profile compared to other ARBs, and there are few studies examining telmisartan/CCB combinations in hypertensive patients [42,15].

In Fogari et al. [42] study, 40 mg of telmisartan and 2.5 mg of amlodipine combination was used. After 4 weeks patients whose BP was not controlled (BP > 130/80 mm Hg) were randomized to two-dose titration regimens, one based on increasing doses of telmisartan (up to 160 mg daily) and fixed 2.5-mg dose of amlodipine, the other based on increasing doses of amlodipine (up to 10 mg daily) and fixed 40-mg dose of telmisartan. It was found that at comparable levels of BP reduction, urinary albumin excretion rate decreased more in subjects treated with escalating doses of telmisartan. Overall, among the different combinations of telmisartan and amlodipine, it is clear that telmisartan 80 mg plus amlodipine 10 mg is the most effective combination and the telmisartan and amlodipine combinations offer a very effective and tolerable option particularly in susceptible patients that require combination therapy.

### ACEIs with ARB

An ACEI/ARB regimen theoretically may provide the advantage of a more complete blockade of the RAAS. ARB will reduce the ACEI escape phenomenon, a mechanism whereby angiotensin II returns to pretreatment levels despite continuous ACEI treatment. Furthermore, angiotensin II generated by ACEI-independent pathways will get blocked by ARBs. Additionally the ACEI itself inhibits bradykinin degradation [43].

Clinical studies of properly dosed ACEI and ARB combinations have demonstrated significant improvement with regard to target organ damage, specifically heart failure and proteinuria. The first major study in this area was the CALM (Candesartan and Lisinopril Microalbuminuria) trial, which was designed to compare the effect of candesartan 16 mg or lisinopril 20 mg or both on BP and urinary albumin-creatinine ratio in 197 type 2 hypertensive microalbuminuric diabetic patients. All three treatments resulted in a significant decrease in both BP and albuminuria. The combination treatment was significantly more effective than monotherapy in reducing BP and resulted in a greater decrease in albuminuria, although this was statistically significant only when the combination was compared with candesartan monotherapy [44].

In the Combination Treatment of angiotensin II receptor blocker and Angiotensin-Converting Enzyme inhibitor in nondiabetic renal Disease (COOPERATE) trial, [45,46] the incidence of a composite renal outcome was reduced by about 60% with combination therapy relative to both monotherapies. However, BP was not lowered to a significantly greater degree than either therapy alone. The Randomized Evaluation of strategies for left Ventricular Dysfunction (RESOLVD) pilot study[47] on patients with heart failure, receiving candesartan, enalapril, or the combination therapy, showed combination therapy to have a more beneficial effect on cardiac volumes and ejection fraction.

However, the potential hazards of ARB plus ACEI combinations must also be taken into consideration: such combinations often produce worsening of hyperkalemia, [48] and may be associated with a decline in the hematocrit in chronic renal failure(CRF) patients with renal anemia [49]. So, the patients receiving this combination treatment should be carefully monitored, particularly in subjects with renal artery stenosis, those receiving concomitant cyclooxygenase inhibitors, or in the elderly, salt depleted, or anemic.

### Comparison of available combinations

Various randomized studies have been conducted to compare fixed combinations of one class with fixed combinations of another class [50-58]. Combinations evaluated are ACEIs/diuretics, ACEIs/CCBs (dihydropyridine and non - dihydropyridine CCBs), β-Adrenoceptor Antagonist/diuretics and ARB/diuretics.

ACEI/CCBs, ACEI/diuretics and β-adrenoceptor antagonist/diuretics all are significantly effective than placebo and helpful in achieving DBP < 90 mm Hg [50]. ACEI/CCBs combination is more effective at reducing both SBP and DBP. ACEI/non dihydropyridine CCBs and ACEI/diuretic have similar efficacy [45,46]. ACEI/dihydropyridine CCBs (amlodipine, manidipine, nitrandipine) combination is more efficacious than ACEI/diuretic combination in reducing both SBP and DBP to a significant extent[47,48]. β-adrenoceptor antagonist/diuretic is similarly effective to ACEI/diuretic and ACEI/CCBs[52]. But β-adrenoceptor antagonist/diuretic have adverse effect on serum lipids and glycemic parameters over one year of treatment [54].

The BP component of ASCOT-BPLA was stopped prematurely after 5.5 years of median follow-up because there was significantly less risk of secondary end points, including nonfatal myocardiac infarction(MI), total cardiovascular end points, all-cause mortality, stroke, and heart failure in patients treated with amlodipine/perindopril compared with those treated with atenolol/bendroflumethiazide. There was also a nonsignificant trend toward reduced risk for the primary end point (nonfatal and fatal MI) favoring amlodipine/perindopril treatment [24]. A subsequent analysis, adjusting for mean BP level, demonstrated reductions of 13% (*P *< .014) and 17% (*P *< .018), respectively, in risks for the primary end point and stroke [55].

In patients with metabolic syndrome, ACEI/CCBs are preferred over β-adrenoceptor antagonist/diuretic and ARB/diuretic combination. ARB/diuretic are associated with marked changes in glucose parameters and a higher incidence of new onset diabetes (26%) as compared to ACEI/CCBs (11%) [56] In patients with Type 2 diabetes although BP reduction is greater with β-adrenoceptor antagonist/diuretic, glycemic control is better stable in patients treated with ACEI/CCBs [57]. In non-diabetic patient, β-adrenoceptor antagonist/diuretic is less effective in reducing DBP compared to ACEI/CCBs (but similar SBP reduction) and had less effect on pulse wave velocity [58].

The ACCOMPLISH trial compared the ACEI benazepril plus the diuretic hydrochlorothiazide (force-titrated to 40/12.5 mg, with the option to raise to 40/25 mg) and benazepril plus amlodipine (force-titrated to 40/5 mg, with the option to raise to 40/10 mg) on a composite cardiovascular mortality and morbidity end point [59]. This combination reduces metabolic disturbances such as hypokalaemia, hyperuricaemia and hypercholesterolaemia, which are all frequent with diuretic monotherapy. The study was terminated prematurely at 36 months because the global cardio vascular disease (CVD) event rate (myocardial infarction, stroke, coronary intervention, heart failure, and other fatal or non fatal cardiovascular disease) diverged early and linearly throughout the trial and was about 19.6% lower (9.6% vs. 11.8%; p,0.001) in those who received amlodipine/benazapril compared to those who received hydrochlorothiazide/benazapril.

ARB/diuretic is similarly effective to ACEI/CCBs in controlling 24 hours BP on ambulatory blood pressure monitoring but less effective in achieving SBP < 140 mm Hg and also associated with poorer metabolic control and new onset diabetes as discussed above [60].

### Some important special situations

#### Metabolic syndrome and Hypertension

##### A) Diabetes and Proteinuria

Hypertension may act synergistically with diabetes in increasing the risk of both macrovascular and microvascular comlplications of diabetes [61]. Various trials, some of which have been randomized, have shown decrease in these complications when BP was lowered to safer limits (< 130/80 mm of Hg). This BP control has been found to be difficult to achieve with monotherapy [62]. Indeed, although ACEIs, ARBs, CCBs, diuretics, and β blockers all have compelling indications in diabetes, it is suggested that combination therapy should include, as initial therapy, an agent that interrupts the RAAS. Second drug can be CCBs or diuretics, or ACEI plus an ARB combination.

The results have consistently shown a beneficial renoprotective effect of ACEIs and ARBs in diabetic nephropathy. The combined therapy with an ARB and a CCB has a potentially useful antiproteinuric effect in patients with type 2 diabetic nephropathy, even when their renal function is reduced. This was also shown in Fogari et al study also [42]. Although treatment with an ARB plus an ACE-I has a greater antiproteinuric effect, but it may be associated with complications including worsening of renal anemia and increased serum potassium concentrations, especially in patients whose kidney function is mildly to moderately impaired.

##### B) Dyslipidemia and Hypertension

Hypertension and dyslipidemia are conditions that can coexist frequently. National Health and Nutrition Examination Survey (NHANES III) has shown that 64% of patients with hypertension also have dyslipidemia and conversely, approximately 47% of patients with dyslipidemia have hypertension. Hypertension and hypercholesterolemia are the two leading risk factors for heart disease, These two together cause an increase in coronary heart disease related events [63].

In addition to its anti-hypertensive effect through antagonizing AT1 receptors, telmisartan has a unique property that activates peroxisome proliferator-activated receptor-γ (PPAR-γ) and is suggested to improve insulin sensitivity and reduce triglyceride levels, leading to a reduction of the risk for atherosclerosis. Miura et al. [64] demonstrated that 12 weeks of treatment with telmisartan (in exchange for valsartan or candesartan) resulted in significant decreases in fasting insulin, fasting blood glucose, hemoglobin A1c and triglycerides; and increases in high density lipoprotein cholesterol and adiponectin, suggesting a potential metabolic and anti-atherogenic benefit.

Saga Telmisartan Aggressive Research (STAR) study evaluated 197 patients being prescribed 20 to 80 mg of telmisartan for 6 months. Total cholesterol (TC) levels decreased from 200 to 188 mg/dl (p < 0.05). Triglyceride levels were decreased 270 to 175 mg/dl (p < 0.005) in patients with TG levels ≥ 150 mg/dl [65]. Telmisartan may accelerate reverse cholesterol transport or inhibit net cholesterol absorption through activation of ABC1, leading to lowering of TC and Low density lipids-Cholesterol [66]. These results suggest that telmisartan may have the ability to lower cholesterol levels, further controlled studies will be needed to confirm these findings. Thus using a telmisartan alone or in combination with a diuretic/CCB can be efficacious in patients with dyslipidemia.

### Heart failure with Hypertension

Treatment of hypertension in patients with heart failure must take into account the type of heart failure, systolic dysfunction or diastolic dysfunction, in which there is a limitation to diastolic filling and therefore in forward output due to increased ventricular stiffness. Diuretics, beta blockers, ACEIs, ARBs, and aldosterone antagonists are indicated in the management of heart failure and have been shown to reduce morbidity and mortality in appropriately selected patients with heart failure. Hyperkalemia could be the side effect of some of these drugs so the drugs like ACEIs, ARBs, and aldosterone antagonist in combination should not be used. The choice of agents is based on severity of heart failure, left ventricular ejection fraction, history of myocardial infarction and any other associated comorbidities.

In these patients, treatment with ACEIs [67] and β-blockers[68] has been shown to improve symptoms and reduce the risk of death and hospitalization for worsening heart failure. β blockers have now become the most extensively studied class of agents in the treatment of Chronic heart failure (CHF), with a database of over 6000 patients in placebo-controlled studies, and ongoing clinical and mechanistic studies. Despite this, further questions remain regarding the use of these agents in CHF, including their role in the extreme elderly, in patients with DM. 2,289 patients with severe CHF in Carvedilol Prospective Randomized Cumulative Survival (COPERNICUS) study, showed improved clinical status and reduced the risk of death with carvedilol as compared to placebo. However, because patients with the lowest SBP were at highest risk of an event, they experienced the greatest absolute benefit from treatment with carvedilol [69].

Angiotensin II type 1 receptor blockers have favourable effects on haemodynamic measurements, neurohumoral activity, and left-ventricular remodelling when added to in patients with ACEIs CHF. The primary outcome of the CHARM- Added study was the composite of cardiovascular death or hospital admission for CHF. Candesartan reduced each of the components of the primary outcome significantly, as well as the total number of hospital admissions for CHF. The benefits of candesartan were similar in all predefined subgroups, including patients receiving baseline beta blocker treatment. The addition of candesartan to ACEI and other treatment leads to a further clinically important reduction in relevant cardiovascular events in patients with CHF and reduced left-ventricular ejection fraction [70].

ONTARGET showed that the ARB telmisartan and the ACEI ramipril are equally effective in preventing cardiovascular events in high-risk patients and that the combination provides no added benefit and causes more adverse effects than either monotherapy in this patient population. However, ONTARGET does not rule out use of the ACEI plus ARB combination in severe heart failure, where angiotensin escape mechanisms are expressed and dual blockade may be needed [71].

### Chronic renal failure with Hypertension

Hypertension can be caused by chronic kidney disease(CKD) but it itself can worsen the renal failure. The guidelines state that management of hypertension in CKD should focus on reducing BP, with some also emphasizing reducing protein excretion.Choice of agent will primarily depend on the presence of proteinuria as there is a direct relationship between the degree of proteinuria and progression to end stage renal disease. In proteinuric kidney first line agents include an ACEIor ARB, and often requires the addition of a diuretic or a calcium channel blocker. Diuretics are a useful alternative for non-proteinuric patients or as an add-on to renin-angiotensin system blockade. Multiple drug therapy is often needed to maintain BP below the 90th percentile target, but adequate BP control is essential for better renal and cardiovascular long-term outcomes. Thiazide diuretics can be used if glomerular filtration rate (GFR) is greater than or equal to 40 mL per minute per 1.73m^2 ^(body surface area), and loop diuretics are used in GFR less than or equal to 40 to 50 mL per minute per 1.73 m^2^[72] Various trials like AASK (African American Study of Kidney Disease), IDNT (Irbesartan in Diabetic Nephropathy Trail), RENAAL (Reduction of Endpoints in Non-insulin dependent diabetes mellitus with the Angiotensin II Antagonist Losartan) show significant risk reduction for CKD progression when proteinuria was reduced by greater than 30% at 6 months.

Recently, combined therapy with an ACEI and an ARB has been shown to provide a greater reduction in urinary albumin excretion (UAE) than monotherapy with either of these agents in patients with diabetic nephropathy. However, such combinations may be associated with potential hazards, including increased serum potassium concentrations and worsening of renal anemia, especially in patients whose kidney function is mildly to moderately impaired [44-47].

Fogari et al. [42] evaluated the effect of a combination therapy with the ARB telmisartan and the long-acting CCB amlodipine on urinary albumin excretion rate(UAER) in hypertensive patients with type 2 diabetes and microalbuminuria. The results of this study demonstrated that the high-dose telmisartan/low-dose amlodipine combination was as effective as low-dose telmisartan/high-dose amlodipine combination in reducing BP values during the 48-week study period without affecting glycemic control or electrolyte plasma levels, but the effect on UAER was significantly more pronounced with the high-dose telmisartan combination, despite equivalent BP-lowering effect [42].

### Hypertension in thyroid disorders

The prevalence of hypertension among patients with hypothyroidism is approximately 3%. Hypertension is much more frequently associated with hyperthyroidism, the prevalence is estimated at 20% to 30%. Hypothyroid state has been shown to accelerate the age-related increases inBP. Studies have shown a significant correlations between DBP and either T4 or T3 suggesting that thyroid hormone deficiency contributes to increase in BP when it is slight to moderate. The mechanism of increased BP in hypothyroidism is not known, but suggested mechanism could be acceleration of structural change of vascular tissue by thyroid hormone deficiency and alteration of autonomic nervous function by thyroid hormone deficiency leading to hemodynamic changes. In patients of thyrotoxicosis, systolic pressures are typically elevated and diastolic pressures are often low, which results in a widened pulse pressure. These findings are attributable to increased cardiac output, stroke volume, heart rate, and cardiac contractility. Although many symptoms of thyrotoxicosis can be controlled with beta-adrenergic blockers, catecholamine levels are usually normal or even decreased. Despite the fact that the activity of the RAAS is increased in patients with thyrotoxicosis, ACEIs and angiotensin II receptor blockers do not always reduceBP. Thus, the role of the RAAS in hypertension associated with thyrotoxicosis remains to be defined.

### Hypertension in elderly population

The estimated prevalence of hypertension in the United States is 66% in men and women aged 60 years and older, which is the highest among all age groups [73]. A metaanalysis showed that treating hypertension in the elderly yields the greatest benefits in relation to stroke (odds ratio, 0.78) and coronary heart disease (odds ratio, 0.75). Importantly, total mortality and coronary heart disease mortality were found to be significantly reduced.^69 ^Treating hypertension in older patients requires attention to their altered physiology and to concomitant cardiovascular and renal disease, which may indicate use of particular antihypertensive drugs. No specific guidelines exist for hypertension management for this particular population. However studies have shown requirement of two or more drugs in most of them. Combination therapy is often necessary to treat isolated systolic hypertension, but control is only reached in 70% of patients in clinical trials following an algorithm [74,75].

Till date, the most encouraging data supporting aggressive management of hypertension in the elderly population comes from the Hypertension In the Very Elderly Trial (HYVET), a randomized, double-blind placebo trial that enrolled 3,845 patients from 195 centers in Europe, China, Australia, and North Africa. Patients were started with indapamide/placebo and were added with perindopril if BP of 150/80 mmHg was not achieved. Fatal stroke, cardiovascular death, heart failure got reduced by 39%, 23% and 64% respectively in a median follow up of 1.8 years. Thus HYVET and other trials favor mono-therapy or combination therapy with thiazide diuretics, ACEIs and CCBs for hypertension in the elderly [76].

### Hypertension in Pregnancy and Breast feeding

Hypertension complicates 5% to 7% of all pregnancies. A subset of preeclampsia, characterized by new-onset hypertension, proteinuria, and multisystem involvement, is responsible for substantial maternal and fetal morbidity and is a marker for future cardiac and metabolic disease [77].

Drugs preferred during the pregnancy are

Ist line - Methyl dopa, Beta blocker (propranolol) and Labetalol

IInd line - Metoprolol, atenolol and Calcium channel blocker (nifedipine)

IIIrd line agents-clonidine, diuretics

Three short acting antihypertensive agents-hydralazine, labetalol, and short acting (sublingual or orally administered) nifedipine-are commonly used to control acute, very high blood pressure in women with severe hypertension in pregnancy.

Maternal antihypertensive drugs usually compatible with breastfeeding are Captopril, diltiazem, Enalapril, Hydralazine, Hydrochlorothiazide, Labetalol, Methyldopa, Minoxidil, beta blockers like Propranolol and timolol, spironolactone and verapamil. Individual side effects of drugs have to be looked for, while prescribing these drugs in lactation.

### Combination of more than 2 drugs

Few patients may require a third or fourth drug to adequately manage BP. Preference should be given to the selection of an agent from a different class than the initial 2 drugs in the combination therapy. Addition of the third drug may be in the form of spironolactone (requires the assessment of renal functions and potassium), minoxidil, hydralazine, carvedilol and rest of the drugs depending on the specific conditions being treated. Centrally acting drugs should be the last option due to potential side effects.

### Contraindications and conditions requiring special care

ACEIs- Pregnancy, angioneurotic edema, hyperkalemia, renal artery stenosis

Diuretics- Gout, Hypokalemia, Pregnancy, Impaired glucose tolerance,

Beta blockers- Asthma, marked bradycardia, abnormal glucose tolerance, obstructive pulmonary disease, peripheral artery disease

ARB- Pregnancy, hyperkalemia, renal artery stenosis

Ca channel blockers- Heart failure, bradyarrythmias

### Concept of "Polypill"

It is generally accepted that reducing the pill burden improves adherence and/or compliance to therapy, though very few data is available to support this theory. Wald and Law introduced the term "polypill" in 2003. Polypill has been thought as a single daily pill to prevent CVD by simultaneously reducing four risk factors (LDL cholesterol, BP, platelet function, and serum homocysteine). It usually is composed of a statin, three pressure-lowering drugs, each at half of its standard dose, aspirin, 75 mg, and folic acid. The polypill was suggested to reduce ischemic heart disease by 88% and stroke by 80% if taken by everyone over 55 years of age [78].

However, our patients present with a puzzle of clinical features, for which variable doses of the specific medication is required. The polypill provides fix combination of substances, possibly resulting in undertreatment of the main condition(s) and overtreatment of secondary conditions. It also neglects differences in metabolism due to age, race and sex. Even after some studies showing its effectiveness the idea is still under investigation and needs to be studied further [79].

## Conclusion

Hypertension is now considered as a part of a complex syndrome of changes in cardiac and vascular structure and function. All of the current guidelines suggest that ≥ 1 antihypertensive agent is required in most patients with hypertension to reach BP goals that will effectively reduce the cardiovascular risk. Therapy with 2 drugs separately or with fixed combinations that include agents with complementary actions. Many combinations have been shown to improve cardiovascular outcome and include a diuretic with the RAAS blocker. Choice of combination therapy depends upon the risk factors, presence of comorbidities like diabetes, renal dysfunction and the adverse effects and tailored according to individual patient.

## List of Abbreviations

BP: Blood Pressure; DM: Diabetes Mellitus; DBP: Diastolic Blood Pressure; JNC: Joint National Committee on Prevention, Detection, Evaluation and Treatment of High Blood Pressure; ESH: European Society of Hypertension; SBP: Systolic Blood Pressure; FDC: Fixed Dose Combinations; HCTZ: Hydrochlorothiazide; ARB: Angiotensin Receptor Blockers; CCB: Calcium Channel Blockers; ACEI: Angiotensin-Converting Enzyme Inhibitor; RAAS: Renin-Angiotensin-Aldosterone System; ASCOT: Anglo-Scandinavian Cardiac Outcomes Trial-Blood Pressure Lowering Arm; CRF: Chronic Renal Failure; MI: Myocardial Infarction; CVD: Cardio Vascular Disease; AT1: Angiotensin II-type 1; TC: Total Cholesterol; CHF: Congestive Heart Failure; CKD: Chronic Kidney Disease; GFR: Glomerular Filteration Rate; UAE: Urinary Albumin Excretion; and UAER: Urinary Albumin Excretion Rate.

## Competing interests

The authors declare that they have no competing interests.

## Authors' contributions

All authors have contributed equally to literature search and paper writing. All authors read and approved the final manuscript
